# The Application of Cognitive Load Theory to the Design of Health and Behavior Change Programs: Principles and Recommendations

**DOI:** 10.1177/10901981251327185

**Published:** 2025-03-27

**Authors:** Kimberley A. Baxter, Nidhi Sachdeva, Sabine Baker

**Affiliations:** 1 Centre for Childhood Nutrition Research, Faculty of Health, Queensland University of Technology, Brisbane, Queensland, Australia; 2 School of Exercise and Nutrition Sciences, Faculty of Health, Queensland University of Technology, Kelvin Grove, Queensland, Australia; 3 Ontario Institute for Studies in Education, University of Toronto, Toronto, Ontario, Canada

**Keywords:** health programs, health literacy, health behavior, cognitive load theory, health equity

## Abstract

Health and behavior change programs play a crucial role in improving health behaviors at individual and family levels. However, these programs face challenges with engagement and retention and typically show modest efficacy. Cognitive load theory is an established and highly used educational theory that proposes individuals have a finite capacity to process new information (“working memory”). Learning, engagement, and performance are negatively impacted when working memory is exceeded. Cognitive load theory is grounded in an understanding of human cognition and conceptualizes different types of cognitive loads imposed on individuals by a learning experience. Cognitive load theory aims to guide the design of learning experiences, considering how the human mind works, leading to more meaningful and effective learning. Cognitive load theory is increasingly applied to domains outside the classroom, such as designing patient and clinical education. Applying cognitive load theory to the design of health programs, their materials, and interfaces can provide insights. By considering the cognitive demands placed on individuals when interacting with health programs, design can be optimized to reduce cognitive load and better facilitate learning and behavior adoption. This may enhance engagement, retention, and effectiveness of programs. Cognitive load theory may be particularly valuable for individuals with diminished working memory due to high levels of mental load and stress. Design principles are presented to consolidate knowledge from cognitive load theory and existing approaches to guide researchers, policymakers, and health programmers. Further research and interdisciplinary collaboration are needed to realize the potential of cognitive load theory in health.

Health and behavior change programs are a cornerstone of health care, used to prevent, manage, and treat acute and long-term health conditions and optimize health and well-being behaviors. These programs are evaluated by their ability to modify behaviors related to an outcome or problem at an individual, community, or population level (Abraham et al., 2009). Unfortunately, promising behavioral programs may encounter challenges translating to real-world settings (Lobb & Colditz, 2013). There is a clear social gradient in health, meaning that people with lower socioeconomic position generally have worse health than those who are more advantaged (Siegrist & Marmot, 2006). Although the burden of poor health is greatest in socio-economically disadvantaged populations, the evidence does not support consistent, positive impacts of health behavior programs in such populations (Karran et al., 2023; Veinot et al., 2018). These populations also face barriers to access related to their structural precarity, such as capacity, time, cost, and cultural misalignment (Gallegos et al., 2023). Understanding how people process new information and the factors that influence this cognitive processing could provide valuable insights into designing health and behavior change programs that aim to modify behaviors.

Cognitive load theory (CLT) is a theory of instructional design based on an understanding of human cognition and working memory (Sweller, 1988). Working memory is the small amount of information that can be held and applied to tasks, whereas long-term memory is the cumulative stored knowledge from one’s life (Cowan, 2014). According to CLT, learning is more effective when cognitive resources are properly managed in working and long-term memory (Sweller et al., 1998). CLT acknowledges the limitation of working memory when dealing with new information, in both capacity and duration. Learning and performance are optimized when instruction is designed according to the human cognitive architecture (Kirschner, 2002).

CLT has been applied extensively in education and instructional design and is gaining recognition within medical and clinical education (Gould et al., 2022; Sewell et al., 2019; Szulewski et al., 2021; Van Merriënboer & Sweller, 2010; Young et al., 2016). It has also been used to a lesser extent in developing health consumer education handouts (Kennedy & Parish, 2021; Wilson & Wolf, 2009) and interventions (Antonio et al., 2023; D. W. Baker et al., 2011). However, there are limited applied examples of CLT in the design of health and behavioral programs or structured principles that could be used across a range of contexts. Health and behavior programs typically incorporate an educational component, requiring individuals to absorb, process, and apply new information and behaviors to a range of situations in their life contexts. By viewing health programs through the lens of “learning,” (i.e., acquiring and applying new knowledge, and to practice long-term memory), CLT can provide ways to design programs, materials, and interfaces that minimize cognitive load and optimize delivery and impact. This article explores the potential of an interdisciplinary approach drawing on CLT and provides future research and practice recommendations. We present the main ideas and implications of CLT and explore the theory’s potential application in the design and development of health and behavior programs. We present connections with emerging knowledge that recognizes the impact of stress on working memory depletion (Chen et al., 2018; Choi et al., 2014), emphasizing the particular value of CLT in designing programs for those experiencing high stress and disadvantage. We offer a series of design principles that consolidate knowledge from CLT and other health theories that can be applied to improve the design of programs, materials, and interfaces that individuals engage with (Feldon et al., 2023).

## Cognitive Load Theory—A Concise Overview

CLT is a scientific approach to designing learning materials and content that considers the limits of working memory. Our working memory is limited in both capacity and duration (Miller, 1956). New information must be processed in working memory before being transferred to be stored in long-term memory. When working memory is exposed to too much information or unnecessary demands, the cognitive load on working memory increases, producing cognitive overload. Learners can feel confused and frustrated when overloaded, and motivation and engagement decrease. However, if working memory is properly managed, there is sufficient capacity for learning to be productive and enjoyable. CLT conceptualizes cognitive load during learning into three elements (Sweller, 2010):

1. *Intrinsic cognitive load* is the task or material’s inherent complexity, the rate of information flow, and the learner’s expertise and prior knowledge. This load is necessary for learning and should be optimized by responding to and adjusting the difficulty level of the learning content for the intended audience.
2. *Extraneous cognitive load* refers to any part of the process that does not facilitate learning. This load is imposed by suboptimal instructional techniques or information presentation and is detrimental to learning. Reducing this load is important and can be achieved by creating clean, simple, and easy-to-follow learning experiences.
3. *Germane cognitive load* is the working memory devoted by the learner to make sense of the new material and store it in long-term memory.

In practice, the more working memory the learner must devote to extraneous load because of poor instructional design, the fewer cognitive resources are available to deal with the intrinsic load of the material, reducing learning (Sweller, 2010). Recently, empirical studies have found that when extraneous cognitive load was reduced, the total cognitive load also decreased (Sweller et al., 2019). Therefore, germane load is not considered an independent source of cognitive load. The key implication of CLT is that instructional strategies and learning materials should optimize intrinsic load because it is helpful for learning while minimizing extraneous load as it impedes learning. Instructional effectiveness will be compromised by the extent to which instructional choices require learners to apply working memory resources to extraneous cognitive load (Sweller, 2010). Germane cognitive load is not an independent load but rather the optimal use of available cognitive capacity after reducing extraneous load.

## Application of CLT to Behavioral Health Programs

Behavior change programs are coordinated sets of activities designed to change specified behavior patterns of individuals, communities, and/or populations through hypothesized or known means (Michie et al., 2011). Programs can be designed to target discreet behaviors or sets of behaviors, such as a parenting approach. In the context of health, behavior change programs can be related to health promotion to prevent disease, manage existing disease, or promote desired practices. Modifiable behaviors for disease prevention might include reducing smoking and alcohol consumption, improving nutrition, managing stress, or increasing physical activity; disease management could consist of medication or therapy adherence programs and health screening (e.g., regular eye checks for diabetes).

Several theoretical models have influenced our understanding of human behavior and informed intervention development. Behavior change theories focus on the complexities of achieving lasting behavior change and can guide program developmsent, implementation, and evaluation. Several behavior change theories can be applied across populations and health contexts (Barley & Lawson, 2016). Some examples include the health belief model (Becker, 1974), the theory of planned behavior (Ajzen, 1991), the social cognitive theory (Bandura, 1986), the transtheoretical model (Prochaska & Velicer, 1997), and the behavior change wheel with the COM-B model (Michie et al., 2011). Each uses a combination of constructs reliant on beliefs, social norms, self-efficacy, and motivation. Digital technologies and increasing community access to such technologies offer opportunities within health to reach individuals in their communities and environments. This has resulted in an upsurge in digital health interventions such as mobile applications, SMS-based programs, and online health websites and platforms (Brewer et al., 2020). From this focus, program development models specific to digital health have also emerged; examples include the IDEAs framework (Mummah et al., 2016) and Behavioral design thinking for mobile health interventions (Voorheis et al., 2022).

These models and frameworks inform the development of a theory of change to explicitly identify the targeted behavior, the mechanisms of how the behavior will be modified, and the expected outcomes. These established models help plan and articulate a program’s what, how, and when. However, health programs face barriers in engaging individuals, typically have modest effects, and are impacted by poor engagement and high attrition (Middleton et al., 2013). CLT provides a framework for developing health materials and aspects of programs that individuals engage with that consider human cognitive factors. By understanding and managing the different types of cognitive loads, programs and materials can be tailored to enhance learning and practice acquisition, recognize potential cognitive loads, and manage them. The benefit of taking an approach informed by CLT may be particularly pronounced for programs aimed at individual behavior change, where an educational or learning component is included, rather than policy or legislative initiatives that aim to influence human behavior through environmental changes or disincentives.

## Contextual Factors

While CLT focuses on the cognitive load directly related to the characteristics of the task, learning task design should not be considered in isolation, as learning occurs within the broader environment and is influenced by contextual factors. Learning cannot happen without the learner, who possesses certain characteristics and is impacted by their environment (Choi et al., 2014). CLT has primarily been applied in educational settings where learning or instruction happens in a controlled environment like a classroom. In real-world settings, the connection between the person and health program is more blurred. The physical environment interacts with the learner’s characteristics, the learning task’s characteristics, or a combination of both (Choi et al., 2014; Jamieson et al., 2000; Tanner, 2008). Affective factors, such as emotional state, stress, and anxiety, are thought to contribute to cognitive load, thereby depleting working memory (de Almeida et al., 2024; Sweller et al., 2019).

For individuals living with disadvantage or poverty, contextual factors may increase cognitive load and impede learning. For example, their home environment may not be conducive to learning (e.g., unsafe, crowded, noisy, or distracting). Managing with limited resources and sporadic income leads to a preoccupation with money and requires individuals to expend cognitive resources on expense management and budgeting (Boswell Dean et al., 2017). A recent qualitative study exploring family food and mealtimes with parents who struggled financially found a common experience was high mental load from the cognitive and emotional work of getting enough food to feed the family with limited economic resources (Baxter, Nambiar, et al., 2024). Poverty may impair cognitive capacity through several pathways, including increased stress, sleep deprivation, hunger, or diminished access to high-quality nutrition, education, and health care (Szaszi et al., 2023).

Another critical consideration in the design of materials and programs is the individual’s health literacy. Health literacy is the personal, cognitive, and social skills that determine one’s ability to gain access to, understand, and use information to promote and maintain good health (Nutbeam, 2000). Low health literacy has been linked to poor understanding of health information and adverse outcomes (Dewalt et al., 2004).

These examples underscore the importance of considering psychosocial and contextual factors that may increase mental load and decrease cognitive capacity. The design of health programs, materials, and interfaces should use strategies that minimize the cognitive load of the program to ensure that social inequalities in health are not further exacerbated. Applying CLT and other cognitive considerations of learning to health and behavioral program design can lead to programs better suited to the cognitive needs of end-users (Pusic et al., 2014).

## Design Principles for the Development of Health and Behavioral Programs

**Figure 1.**
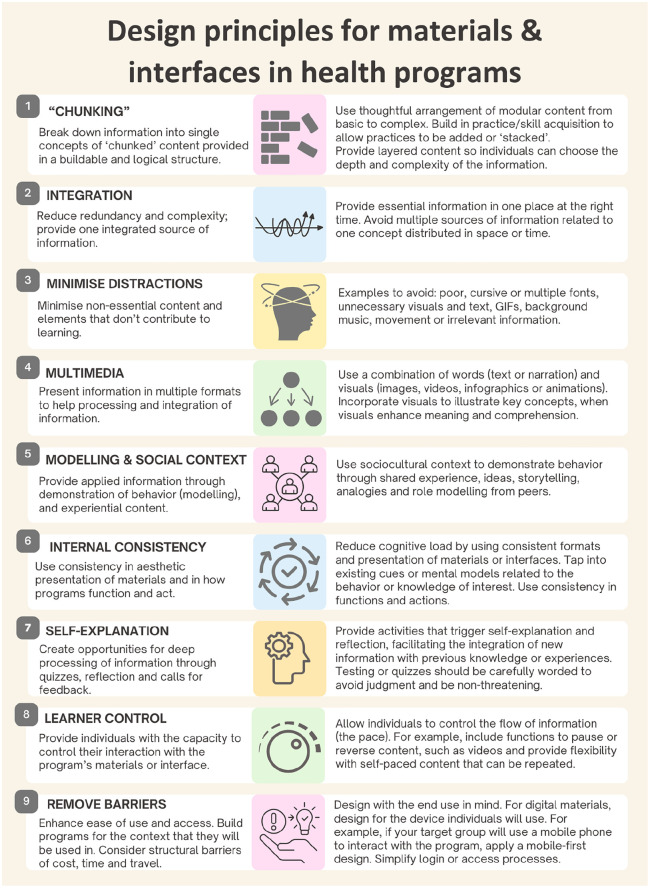
Design Principles for Use in Health and Behavior Change Programs.

Figure 1 summarizes design principles, which are further described in this section to consolidate knowledge from CLT and existing health and behavioral approaches. These design principles aim to organize programs, interfaces, and materials to reduce extraneous cognitive load, thereby directing working memory to learning. Health researchers and program designers can utilize these strategies alongside a plain language approach, content expertise, and health behavior models that inform the program’s theory of change. These principles can be applied holistically or selectively to materials and content development in a way that makes sense for the program.

Plain Language principles are a commonly used and recommended approach in developing health communications, and established guidelines exist for their application (Stableford & Mettger, 2007). Plain Language emphasizes clear, concise language, including simpler words and shorter sentences, without unnecessary complexity and jargon. Studies suggest that using plain language can improve the comprehensibility of complex health information (Grene et al., 2017). Using pictorial aids is also recommended to help understand abstract ideas and complex instructions and is commonly employed for individuals with lower health literacy (Mayer, 2021b; Schubbe et al., 2020).

Developing health programs and materials in alignment with the limitations of working memory can further promote comprehension and recall. Intrinsic load is often overlooked by designers, in part because designers can possess “expert blindness” (i.e., the inability of experts to perceive the kinds of confusion that a novice might have (Nathan et al., 2001)). For any set of educational materials, a learner’s level of intrinsic load is a combination of the material’s inherent complexity and the knowledge an individual possesses. For example, if the educational materials use language unfamiliar to a learner, that person will experience higher levels of intrinsic load. This is important when designing materials for large audiences with varying literacy levels. Thus, one way to reduce intrinsic load is to use simple language and, where possible, to augment written or verbal information with clear, easy-to-understand images and animations.

Another strategy for reducing intrinsic load is to reduce the inherent complexity of the instruction. This can be accomplished by chunking new information and presenting the material bit by bit (Paas et al., 2004). Effective chunking requires the designer to think carefully about the size and sequencing of each chunk of information to ensure that prerequisite knowledge is taught before more advanced knowledge. Providing examples, stories, scenarios, and models contextualizes and applies information. Designers can minimize the extraneous load within educational materials by eliminating unnecessary information, eliminating ambiguities, using clear language, and removing visual elements that are strictly decorative (Castro-Alonso et al., 2021). Reducing extraneous load can be achieved by reducing unnecessary complexity in health information and programs, thus freeing up cognitive resources for processing essential information (Kalyuga & Sweller, 2021).

Learning can be encouraged by designing materials that promote the integration of new information with existing knowledge, facilitating the embedding of knowledge and practice in long-term memory. One way this can be encouraged is by providing opportunities for individuals to reflect on the content and provide feedback on the materials; this scaffolds opportunities to make connections and organize information. This is especially important in health programs as behaviors happen within an individual’s broader life; therefore, addressing previous behaviors and knowledge and integrating them with new practices are essential to long-term behavior change.

Another consideration when developing health and behavioral programs is the cognitive theory of multimedia learning (CTML) (Mayer, 2021a). Based on cognitive load and information processing theories, it proposes a series of evidence-based multimedia principles to design content that promotes active learning, wherein the learner can select information, organize it, and integrate it with previous knowledge. Some of these multimedia principles that can inform the design of health programs include the *multimedia principle* (to present words and pictures rather than words alone), *spatial contiguity principle* (to present text and corresponding graphics close to one another), *coherence principle* (to remove distracting content from materials), and *segmenting principle* (to divide the content into segments, so as not to overload working memory). While generally useful for designing learning content, multimedia principles are especially beneficial for developing digital content.

## Exemplary Approaches Applying CLT

Techniques informed by CLT have been successfully applied to specific settings where integrating knowledge sets and practical skills is essential. Such an example is a multisession education program for heart failure management among low literacy patients (D. W. Baker et al., 2011). This research described design principles for the program informed by cognitive load and learning mastery theory to improve the design of education curricula and programs (D. W. Baker et al., 2011). Another cognitive theory-informed approach is microlearning, which is gaining significant traction in clinical training and health settings (De Gagne et al., 2019). Microlearning refers to small lesson modules and activities that focus on discrete concepts and can take many forms, including web-based applications, mobile gaming devices, and videos. The key feature of microlearning is the small content size, which can be completed in short bursts (Torgerson, 2016). The asynchronous nature of microlearning allows the individual to control the place, time, and pace of the learning interaction and applies well to digital formats and platforms. Microlearning in health professional training has been found to have benefits for knowledge retention, effectiveness, and increased engagement (De Gagne et al., 2019). In parenting support settings, microlearning has been identified to address barriers, such as the logistics of attending face-to-face sessions, declining enrolment, high attrition, and lower uptake in socioeconomic and racial groups (Grodberg & Smith, 2022). Digital modes for parenting programs offer an opportunity to reach a broad range of parents, including higher-risk families (S. Baker et al., 2017). A recent study that developed a child-feeding program identified digital microlearning as a mode and delivery method aligned with the needs of low-income families who experienced high stress and food insecurity (Baxter, Kerr, et al., 2024). It has also been shown in a systematic review that microlearning can improve an individual’s self-care capability, including health-related self-care and health literacy (Wang et al., 2020). Approaches that consider CLT, offer opportunities within health and behavior programs that address some of the barriers and challenges associated with traditional techniques and methods of delivery.

## Recommendations and Implications for Practice and Research

Innovative approaches are needed to improve engagement and outcomes in health programs and offer new perspectives. Researchers and program developers can gain insights from educational theory that considers the learning process and cognitive factors. CLT provides a promising framework for optimizing health programs, materials, and interfaces. Central to CLT is the recognition that individuals have finite cognitive resources, which can be overwhelmed by complex information, environmental distractions, and affective factors such as stress. For populations experiencing disadvantage and poverty, considerations of reduced mental resources are often compounded by systemic barriers to health program access, lower literacy levels, and higher experiences of psychosocial stress (Evans & Kim, 2013). Research suggests that individuals experiencing poverty and social disadvantage are more likely to face chronic stressors related to financial strain, housing instability, and exposure to family conflict and tension, which contribute to elevated levels of cognitive load (Mani et al., 2013). In addition, lower literacy levels among individuals may be partly explained by cognitive effects, such as reduced working memory (Federman et al., 2009; Wilson et al., 2010). This calls for the design of materials and programs that take cognitive factors such as working memory into account beyond considering readability and simplifying language. Designing health programs and materials in ways that optimize intrinsic load and reduce extraneous load can reduce the cognitive demands placed on individuals, thereby enhancing accessibility, engagement, and effectiveness.

It is important to recognize that within educational theory, critical opinions exist on the methodological rigor and conceptual clarity of CLT and the delineation of the loads proposed by CLT (de Jong, 2010; Martin, 2018). Further advances in measuring intrinsic and extraneous loads are needed to empirically test theoretical explanations of the effects of manipulating instructional designs (Schnotz & Kürschner, 2007). However, the assertion that individuals have a limited working memory is a central tenet of education, and it is widely accepted that the design of instruction impacts the learning experience. For this article, we propose that an understanding of the concepts of intrinsic and extraneous cognitive load provides a framework that can be used to optimize modifiable design elements. Furthermore, we have presented connections with the complex factors surrounding individuals, such as disadvantage, low resources, and low literacy, which creates an imperative to design materials as effectively as possible and consider the limits of individuals’ working memory.

The process of developing health program materials and interfaces should consider cognitive factors. Intrinsic and extrinsic loads place cognitive demands on individuals, which can be reduced through thoughtful design, such as the principles and strategies presented in this article. Optimizing these loads through well-designed programs and materials will provide the best opportunity to support, engage, and retain individuals in promoting behaviors for positive health change. This may improve long-term learning and behavior adoption, leading to better outcomes and higher satisfaction and engagement. Moving forward, further research and interdisciplinary collaboration are needed to fully realize the potential of CLT to enhance approaches to health and behavioral program design.

## References

[bibr1-10901981251327185] Abraham C. Kelly M. P. West R. Michie S. (2009). The UK National Institute for Health and Clinical Excellence public health guidance on behaviour change: A brief introduction. Psychology, Health & Medicine, 14(1), 1–8. 10.1080/13548500802537903 19085307

[bibr2-10901981251327185] Ajzen I. (1991). The theory of planned behavior. Organizational Behavior and Human Decision Processes, 50(2), 179–211. 10.1016/0749-5978(91)90020-T

[bibr3-10901981251327185] Antonio M. G. Williamson A. Kameswaran V. Beals A. Ankrah E. Goulet S. Wang Y. Macias G. James-Gist J. Brown L. K. Davis S. Pillai S. Buis L. Dillahunt T. Veinot T. C. (2023). Targeting patients’ cognitive load for telehealth video visits through student-delivered helping sessions at a United States Federally Qualified Health Center: Equity-focused, mixed methods pilot intervention study [Original paper]. Journal of Medical Internet Research, 25, Article e42586. 10.2196/42586 PMC9897309 36525332

[bibr4-10901981251327185] Baker D. W. DeWalt D. A. Schillinger D. Hawk V. Ruo B. Bibbins-Domingo K. Weinberger M. Macabasco-O’Connell A. Pignone M. (2011). “Teach to goal”: theory and design principles of an intervention to improve heart failure self-management skills of patients with low health literacy. Journal of Health Communication, 16(Suppl. 3), 73–88. 10.1080/10810730.2011.604379 21951244 PMC3454452

[bibr5-10901981251327185] Baker S. Sanders M. R. Morawska A. (2017). Who uses online parenting support? A cross-sectional survey exploring Australian parents’ Internet use for parenting. Journal of Child and Family Studies, 26(3), 916–927. 10.1007/s10826-016-0608-1

[bibr6-10901981251327185] Bandura A . (1986). Social foundations of thought and action: A social cognitive theory. Prentice-Hall, Inc.

[bibr7-10901981251327185] Barley E. Lawson V. (2016). Using health psychology to help patients: Theories of behaviour change. British Journal of Nursing, 25(16), 924–927. 10.12968/bjon.2016.25.16.924 27615529

[bibr8-10901981251327185] Baxter K. A. Kerr J. Nambiar S. Gallegos D. Penny R. A. Laws R. Byrne R. (2024). A design thinking-led approach to develop a responsive feeding intervention for Australian families vulnerable to food insecurity: Eat, Learn, Grow. Health Expectations, 27(2), Article e14051. 10.1111/hex.14051 PMC11032130 38642335

[bibr9-10901981251327185] Baxter K. A. Nambiar S. Penny R. Gallegos D. Byrne R. (2024). Food insecurity and feeding experiences among parents of young children in Australia: An exploratory qualitative study. Journal of the Academy of Nutrition and Dietetics, 124, 1277–1287. 10.1016/j.jand.2024.02.016 38428454

[bibr10-10901981251327185] Becker M. H. (1974). The health belief model and sick role behavior. Health Education Monographs, 2(4), 409–419. 10.1177/109019817400200407

[bibr11-10901981251327185] Boswell Dean E. Schilbach F. Schofield H . (2017). Poverty and cognitive function. University of Chicago Press. 10.7208/chicago/9780226574448.001.0001

[bibr12-10901981251327185] Brewer L. C. Fortuna K. L. Jones C. Walker R. Hayes S. N. Patten C. A. Cooper L. A. (2020). Back to the future: Achieving health equity through health informatics and digital health. JMIR mHealth uHealth, 8(1), Article e14512. 10.2196/14512 PMC6996775 31934874

[bibr13-10901981251327185] Castro-Alonso J. C. de Koning B. B. Fiorella L. Paas F. (2021). Five strategies for optimizing instructional materials: Instructor- and learner-managed cognitive load. Educational Psychology Review, 33(4), 1379–1407. 10.1007/s10648-021-09606-9 33716467 PMC7940870

[bibr14-10901981251327185] Chen O. Castro-Alonso J. C. Paas F. Sweller J. (2018). Extending cognitive load theory to incorporate working memory resource depletion: Evidence from the spacing effect. Educational Psychology Review, 30(2), 483–501. 10.1007/s10648-017-9426-2

[bibr15-10901981251327185] Choi H. H. van Merriënboer J. J. G. Paas F. (2014). Effects of the physical environment on cognitive load and learning: Towards a new model of cognitive load. Educational Psychology Review, 26(2), 225–244. 10.1007/s10648-014-9262-6

[bibr16-10901981251327185] Cowan N. (2014). Working memory underpins cognitive development, learning, and education. Educational Psychology Review, 26(2), 197–223. 10.1007/s10648-013-9246-y 25346585 PMC4207727

[bibr17-10901981251327185] de Almeida F. Scott I. J. Soro J. C. Fernandes D. Amaral A. R. Catarino M. L. Arêde A. Ferreira M. B . (2024). Financial scarcity and cognitive performance: A meta-analysis. Journal of Economic Psychology, 101, 102702. 10.1016/j.joep.2024.102702

[bibr18-10901981251327185] De Gagne J. C. Park H. K. Hall K. Woodward A. Yamane S. Kim S. S . (2019). Microlearning in health professions education: Scoping review. JMIR Medical Education, 5(2), Article e13997. 10.2196/13997 PMC6683654 31339105

[bibr19-10901981251327185] de Jong T . (2010). Cognitive load theory, educational research, and instructional design: Some food for thought. Instructional Science, 38(2), 105–134. 10.1007/s11251-009-9110-0

[bibr20-10901981251327185] Dewalt D. A. Berkman N. D. Sheridan S. Lohr K. N. Pignone M. P. (2004). Literacy and health outcomes: A systematic review of the literature. Journal of General Internal Medicine, 19(12), 1228–1239. 10.1111/j.1525-1497.2004.40153.x 15610334 PMC1492599

[bibr21-10901981251327185] Evans G. W. Kim P. (2013). Childhood poverty, chronic stress, self-regulation, and coping. Child Development Perspectives, 7(1), 43–48. 10.1111/cdep.12013

[bibr22-10901981251327185] Federman A. D. Sano M. Wolf M. S. Siu A. L. Halm E. A. (2009). Health literacy and cognitive performance in older adults. Journal of the American Geriatrics Society, 57(8), 1475–1480. 10.1111/j.1532-5415.2009.02347.x 19515101 PMC2754116

[bibr23-10901981251327185] Feldon D. F. Brockbank R. Litson K. (2023). Direct effects of cognitive load on self-efficacy during instruction. Journal of Educational Psychology, 116(7), 1153–1174. 10.1037/edu0000826

[bibr24-10901981251327185] Gallegos D. Durham J. Rutter C. McKechnie R. (2023). Working towards the active participation of underrepresented populations in research: A scoping review and thematic synthesis. Health & Social Care in the Community, 2023, 1312525. 10.1155/2023/1312525

[bibr25-10901981251327185] Gould D. J. Sawarynski K. Mohiyeddini C. (2022). Academic management in uncertain times: Shifting and expanding the focus of cognitive load theory during COVID-19 pandemic education. Frontiers in Psychology, 13, Article 647904. 10.3389/fpsyg.2022.647904 PMC9249437 35783760

[bibr26-10901981251327185] Grene M. Cleary Y. Marcus-Quinn A. (2017). Use of plain-language guidelines to promote health literacy. IEEE Transactions on Professional Communication, 60(4), 384–400. 10.1109/TPC.2017.2761578

[bibr27-10901981251327185] Grodberg D. Smith I. (2022). Scaling parent management training through digital and microlearning approaches. Frontiers in Psychology, 13, Article 934665. 10.3389/fpsyg.2022.934665 PMC9534294 36211919

[bibr28-10901981251327185] Jamieson P. Fisher K. Gilding T. Taylor P. Trevitt A. C. F. (2000). Place and space in the design of new learning environments. Higher Education Research & Development, 19, 221–236.

[bibr29-10901981251327185] Kalyuga S. Sweller J. (2021). The redundancy principle in multimedia learning. In Mayer R. E. Fiorella L. (Eds.), The Cambridge handbook of multimedia learning (3rd ed., pp. 212–220). Cambridge University Press. 10.1017/9781108894333.021

[bibr30-10901981251327185] Karran E. L. Grant A. R. Lee H. Kamper S. J. Williams C. M. Wiles L. K. Shala R. Poddar C. V. Astill T. Moseley G. L. (2023). Do health education initiatives assist socioeconomically disadvantaged populations? A systematic review and meta-analyses. BMC Public Health, 23(1), Article 453. 10.1186/s12889-023-15329-z PMC9996883 36890466

[bibr31-10901981251327185] Kennedy M. B. Parish A. L. (2021). Educational theory and cognitive science: Practical principles to improve patient education. Nursing Clinics of North America, 56(3), 401–412. 10.1016/j.cnur.2021.04.006 34366160

[bibr32-10901981251327185] Kirschner P. A. (2002). Cognitive load theory: Implications of cognitive load theory on the design of learning. Learning and Instruction, 12(1), 1–10. 10.1016/S0959-4752(01)00014-7

[bibr33-10901981251327185] Lobb R. Colditz G. A. (2013). Implementation science and its application to population health. Annual Review of Public Health, 34, 235–251. 10.1146/annurev-publhealth-031912-114444 PMC3901430 23297655

[bibr34-10901981251327185] Mani A. Mullainathan S. Shafir E. Zhao J. (2013). Poverty impedes cognitive function. Science, 341(6149), 976–980. 10.1126/science.1238041 23990553

[bibr35-10901981251327185] Martin S. (2018). A critical analysis of the theoretical construction and empirical measurement of cognitive load. In Zheng R. Z. (Ed.), Cognitive load measurement and application: A theoretical framework for meaningful research and practice (pp. 29–44). Routledge, Taylor & Francis Group. 10.4324/9781315296258-3

[bibr36-10901981251327185] Mayer R. E. (2021a). Cognitive theory of multimedia learning. In Mayer R. E. Fiorella L. (Eds.), The Cambridge handbook of multimedia learning (3rd ed., pp. 57–72). Cambridge University Press. 10.1017/9781108894333.008

[bibr37-10901981251327185] Mayer R. E. (2021b). The multimedia principle. In Mayer R. E. Fiorella L. (Eds.), The Cambridge handbook of multimedia learning (3rd ed., pp. 145–157). Cambridge University Press. 10.1017/9781108894333.015

[bibr38-10901981251327185] Michie S. van Stralen M. M. West R. (2011). The behaviour change wheel: A new method for characterising and designing behaviour change interventions. Implementation Science, 6(1), 42. 10.1186/1748-5908-6-42 21513547 PMC3096582

[bibr39-10901981251327185] Middleton K. R. Anton S. D. Perri M. G. (2013). Long-term adherence to health behavior change. American Journal of Lifestyle Medicine, 7(6), 395–404. 10.1177/1559827613488867 27547170 PMC4988401

[bibr40-10901981251327185] Miller G. A. (1956). The magical number seven plus or minus two: Some limits on our capacity for processing information. Psychological Review, 63(2), 81–97. 13310704

[bibr41-10901981251327185] Mummah S. A. Robinson T. N. King A. C. Gardner C. D. Sutton S. (2016). IDEAS (Integrate, Design, Assess, and Share): A framework and toolkit of strategies for the development of more effective digital interventions to change health behavior [Viewpoint]. Journal of Medical Internet Research, 18(12), Article e317. 10.2196/jmir.5927 PMC5203679 27986647

[bibr42-10901981251327185] Nathan M. J. Koedinger K. Alibali M. W. (2001). Expert blind spot: When content knowledge eclipses pedagogical content knowledge. In Proceedings of the Third International Conference on Cognitive Science (pp. 644–648).

[bibr43-10901981251327185] Nutbeam D. (2000). Health literacy as a public health goal: A challenge for contemporary health education and communication strategies into the 21st century. Health Promotion International, 15(3), 259–267. 10.1093/heapro/15.3.259

[bibr44-10901981251327185] Paas F. Renkl A. Sweller J. (2004). Cognitive load theory: Instructional implications of the interaction between information structures and cognitive architecture. Instructional Science, 32(1/2), 1–8. http://www.jstor.org/stable/41953634

[bibr45-10901981251327185] Prochaska J. O. Velicer W. F. (1997). The transtheoretical model of health behavior change. American Journal of Health Promotion, 12(1), 38–48. 10.4278/0890-1171-12.1.38 10170434

[bibr46-10901981251327185] Pusic M. V. Ching K. Yin H. S. Kessler D. (2014). Seven practical principles for improving patient education: Evidence-based ideas from cognition science. Paediatrics & Child Health, 19(3), 119–122. 10.1093/pch/19.3.119 24665218 PMC3959967

[bibr47-10901981251327185] Schnotz W. Kürschner C. (2007). A reconsideration of cognitive load theory. Educational Psychology Review, 19(4), 469–508. 10.1007/s10648-007-9053-4

[bibr48-10901981251327185] Schubbe D. Scalia P. Yen R. W. Saunders C. H. Cohen S. Elwyn G. van den Muijsenbergh M. Durand M. A. (2020). Using pictures to convey health information: A systematic review and meta-analysis of the effects on patient and consumer health behaviors and outcomes. Patient Education and Counseling, 103(10), 1935–1960. 10.1016/j.pec.2020.04.010 32466864

[bibr49-10901981251327185] Sewell J. L. Maggio L. A. ten Cate O. van Gog T. Young J. Q. O’Sullivan P. S. (2019). Cognitive load theory for training health professionals in the workplace: A BEME review of studies among diverse professions: BEME guide no. 53. Medical Teacher, 41(3), 256–270. 10.1080/0142159x.2018.1505034 30328761

[bibr50-10901981251327185] Siegrist J. Marmot M. (2006). Social Inequalities in Health: New evidence and policy implications. Oxford University Press. 10.1093/acprof:oso/9780198568162.001.0001

[bibr51-10901981251327185] Stableford S. Mettger W. (2007). Plain language: A strategic response to the health literacy challenge. Journal of Public Health Policy, 28(1), 71–93. 10.1057/palgrave.jphp.3200102 17363939

[bibr52-10901981251327185] Sweller J. (1988). Cognitive load during problem solving: Effects on learning. Cognitive Science, 12, 257–285. https://api.semanticscholar.org/CorpusID:9585835

[bibr53-10901981251327185] Sweller J. (2010). Element interactivity and intrinsic, extraneous, and germane cognitive load. Educational Psychology Review, 22(2), 123–138. 10.1007/s10648-010-9128-5

[bibr54-10901981251327185] Sweller J. van Merriënboer J. J. G. Paas F. (2019). Cognitive architecture and instructional design: 20 years later. Educational Psychology Review, 31(2), 261–292. 10.1007/s10648-019-09465-5

[bibr55-10901981251327185] Sweller J. van Merrienboer J. J. G. Paas F. G. W. C . (1998). Cognitive architecture and instructional design. Educational Psychology Review, 10(3), 251–296. 10.1023/a:1022193728205

[bibr56-10901981251327185] Szaszi B. Palfi B. Neszveda G. Taka A. Szécsi P. Blattman C. Jamison J. C. Sheridan M. (2023). Does alleviating poverty increase cognitive performance? Short- and long-term evidence from a randomized controlled trial. Cortex, 169, 81–94. 10.1016/j.cortex.2023.07.009 37866061

[bibr57-10901981251327185] Szulewski A. Howes D. van Merriënboer J. J. G. Sweller J. (2021). From theory to practice: The application of cognitive load theory to the practice of medicine. Academic Medicine, 96(1), 24–30. 10.1097/acm.0000000000003524 32496287

[bibr58-10901981251327185] Tanner C. K. (2008). Explaining relationships among student outcomes and the school’s physical environment. Journal of Advanced Academics, 19(3), 444–471. 10.4219/jaa-2008-812

[bibr59-10901981251327185] Torgerson C. (2016). The microlearning guide to microlearning. Torgerson Consulting. https://books.google.com.au/books?id=i_1EMQAACAAJ

[bibr60-10901981251327185] Van Merriënboer J. J. G. Sweller J . (2010). Cognitive load theory in health professional education: Design principles and strategies. Medical Education, 44(1), 85–93. 10.1111/j.1365-2923.2009.03498.x 20078759

[bibr61-10901981251327185] Veinot T. C. Mitchell H. Ancker J. S. (2018). Good intentions are not enough: How informatics interventions can worsen inequality. Journal of the American Medical Informatics Association, 25(8), 1080–1088. 10.1093/jamia/ocy052 29788380 PMC7646885

[bibr62-10901981251327185] Voorheis P. Zhao A. Kuluski K. Pham Q. Scott T. Sztur P. Khanna N. Ibrahim M. Petch J. (2022). Integrating behavioral science and design thinking to develop mobile health interventions: Systematic scoping review [Review]. JMIR mHealth uHealth, 10(3), Article e35799. 10.2196/35799 PMC8968622 35293871

[bibr63-10901981251327185] Wang C. Bakhet M. Roberts D. Gnani S. El-Osta A. (2020). The efficacy of microlearning in improving self-care capability: A systematic review of the literature. Public Health, 186, 286–296. 10.1016/j.puhe.2020.07.007 32882481

[bibr64-10901981251327185] Wilson E. A. H. Wolf M. S. (2009). Working memory and the design of health materials: A cognitive factors perspective. Patient Education and Counseling, 74(3), 318–322. 10.1016/j.pec.2008.11.005 19121915

[bibr65-10901981251327185] Wilson E. A. H. Wolf M. S. Curtis L. M. Clayman M. L. Cameron K. A. Eigen K. V. Makoul G. (2010). Literacy, cognitive ability, and the retention of health-related information about colorectal cancer screening. Journal of Health Communication, 15(Suppl. 2), 116–125. 10.1080/10810730.2010.499984 20845198

[bibr66-10901981251327185] Young J. Q. ten Cate O. O’Sullivan P. S. Irby D. M. (2016). Unpacking the complexity of patient handoffs through the lens of cognitive load theory. Teaching and Learning in Medicine, 28(1), 88–96. 10.1080/10401334.2015.1107491 26787089

